# Supplementary material for: Ecotype-Specific and Correlated Seasonal Responses of Biomass Production, Non-Structural Carbohydrates, and Fatty Acids in Zostera marina
Source: Plants (Basel). 2024 Jan 29;13(3):396. doi: 10.3390/plants13030396 (PMC10856944; doi:10.3390/plants13030396)
Supplement: Supplementary file 1 [file plants-13-00396-s001.zip › plants-2792317-supplementary.pdf]

Supplementary material

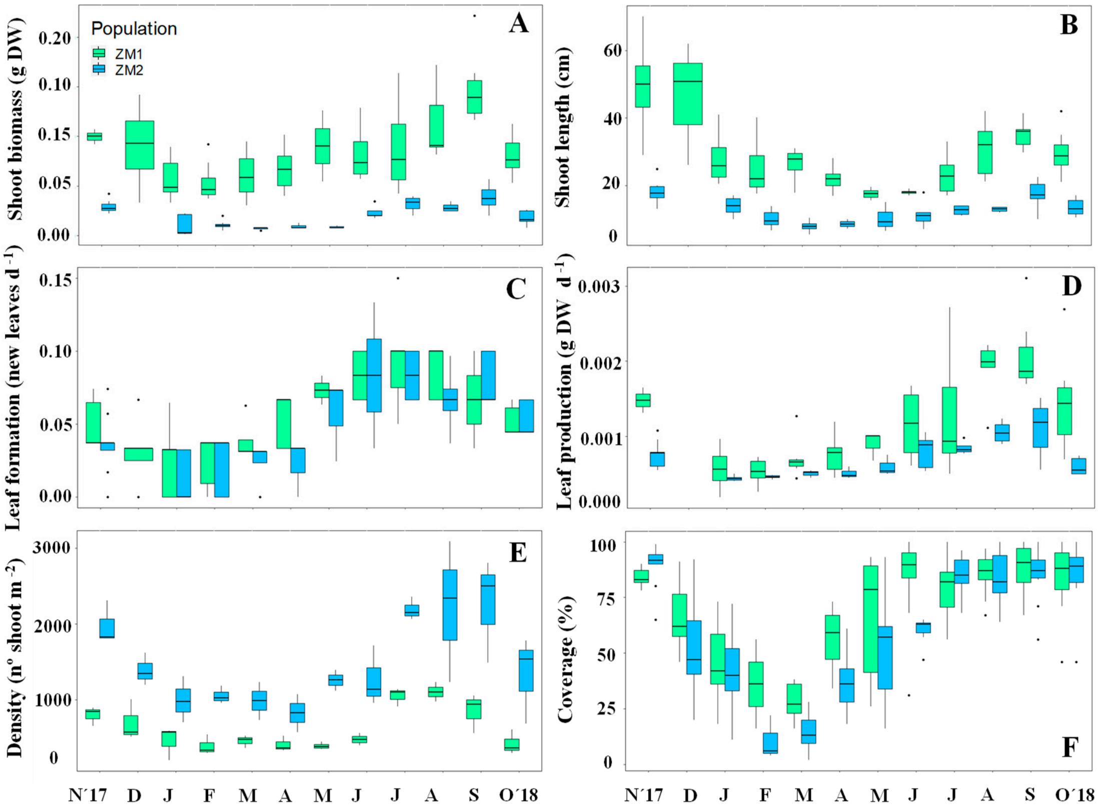

Figure S1: Seasonal trends of: (A) shoot biomass (g DW shoot $^{-1}$); (B) shoot length (cm); (C) leaf formation (new leaves d^{-1}); (D) leaf biomass production (g DW d^{-1} shoot $^{-1}$); (E) shoot density (nº shoot m^{-2}); and (F) coverage (%) of *Zostera marina* from Ireland. Data represented as mean ± SD (n=3-13). For ZM1, data was extracted from Azcárate-García et al. (2022). Black points are considered outliers.

Table S1. Monthly results of mean shoot biomass, mean shoot length, mean leaf formation, mean leaf production, mean density and mean cover of two Irish ecotypes of *Zostera marina* (ZM1 and ZM2). Data represented as mean ± SD (n=3-13). For ZM1, data was extracted from Azcárate-García et al. (2022).

**Month**	**Ecotype**	**Shoot biomass (g DW shoot^{-1})**	**Shoot length (cm)**	**Leaf formation (N° of new leaves d^{-1})**	**Leaf biomass production (g DW d^{-1} shoot^{-1})**	**Density (shoot m^{-2})**	**Cover (%)**
**Nov**	ZM1	0.10 ± 0.0061	29.75 ± 0.61	0.048 ± 0.017	0.0015 ± 0.00014	800.00± 103.6	84.25 ± 3.54
	ZM2	0.029 ± 0.0057	18.09 ± 3.02	0.033 ± 0.020	0.00075 ± 0.00016	1978.67 ± 230.1	89.75 ± 8.74
**Dec**	ZM1	0.16 ± 0.086	46.51 ± 12.39	0.029 ± 0.020	-	698.67 ± 220.2	66.91 ± 13.44
	ZM2	-	-	-	-	1386.67 ± 172.4	51.27 ± 18.38
**Jan**	ZM1	0.056 ± 0.018	27.7 ± 6.82	0.023 ± 0.021	0.00057 ± 0.00022	458.67 ± 177.37	44.86 ± 15.01
	ZM2	0.010 ± 0.0097	13.91 ± 2.54	0.014 ± 0.016	0.00044 ± 0.00003	997.33 ± 248.67	41.13 ± 18.95
**Feb**	ZM1	0.053 ± 0.017	25.63 ± 7.87	0.026 ± 0.017	0.00054 ± 0.00014	394.67 ± 106.40	36.00 ± 16.33
	ZM2	0.011 ± 0.0043	10.10 ± 2.52	0.021 ± 0.018	0.00046 ± 0.00002	1056.00 ± 94.21	10.67 ± 8.06
**Mar**	ZM1	0.061 ± 0.021	26.49 ± 4.09	0.039 ± 0.014	0.00070 ± 0.00023	458.67 ± 67.04	28.22 ± 7.39
	ZM2	0.007 ± 0.0011	7.90 ± 1.74	0.023 ± 0.013	0.00050 ± 0.00004	986.67 ± 202.53	14.50 ± 7.62
**Apr**	ZM1	0.067 ± 0.020	21.98 ± 3.44	0.052 ± 0.017	0.00076 ± 0.00023	410.67 ± 83.99	57.00 ± 11.75
	ZM2	0.009 ± 0.0023	8.57 ± 1.14	0.022 ± 0.016	0.00051 ± 0.00007	826.67 ± 202.53	35.38 ± 11.53
**May**	ZM1	0.090 ± 0.029	17.50 ± 1.63	0.073 ± 0.000	0.00090 ± 0.00016	394.67 ± 39.91	66.10 ± 25.05
	ZM2	0.008 ± 0.0012	10.23 ± 3.55	0.057 ± 0.023	0.00060 ± 0.00011	1258.67 ± 111.11	51.10 ± 22.53
**Jun**	ZM1	0.083 ± 0.028	18.00 ± 0.71	0.083 ± 0.017	0.0012 ± 0.00044	480.00 ± 65.32	84.58 ± 18.23
	ZM2	0.023 ± 0.0061	11.50 ± 3.66	0.083 ± 0.037	0.00080 ± 0.0002	1269.33 ± 321.15	60.00 ± 5.83
**Jul**	ZM1	0.089 ± 0.042	23.25 ± 5.64	0.094 ± 0.012	0.0013 ± 0.00077	1050.67 ± 98.92	79.30 ± 13.09
	ZM2	0.031 ± 0.0077	12.63 ± 1.38	0.083 ± 0.017	0.00085 ± 0.00008	2186.67 ± 121.38	85.10 ± 7.97
**Aug**	ZM1	0.11 ± 0.034	30.92 ± 7.76	0.087 ± 0.016	0.0019 ± 0.00039	1104.00 ± 104.51	85.67 ± 10.66
	ZM2	0.028 ± 0.0041	12.90 ± 0.70	0.067 ± 0.000	0.0011 ± 0.00014	2218.67 ± 762.24	84.20 ± 10.73
**Sept**	ZM1	0.15 ± 0.034	34.94 ± 3.76	0.067 ± 0.025	0.0021 ± 0.00047	853.33 ± 212.40	88.75 ± 9.55
	ZM2	0.038 ± 0.011	17.47 ± 3.67	0.080 ± 0.016	0.0011 ± 0.0003	2261.33 ± 560.74	84.60 ± 11.93
**Oct**	ZM1	0.080 ± 0.018	29.60 ± 5.70	0.051 ± 0.010	0.0014 ± 0.00054	426.67 ±130.86	84.64 ± 15.32
	ZM2	0.018 ± 0.0069	13.52 ± 2.41	0.053 ± 0.011	0.00060 ± 0.0001	1333.33 ± 466.72	84.80 ± 14.34

Table S2. Monthly results of mean total leaf area, leaf production and leaf area index of two Irish ecotypes of *Zostera marina* (ZM1 and ZM2). Data represented as mean ± SD (n=3-13).

	Ecotype	Total leaf area (cm^2 $shoot^{-1}$)	Leaf biomass production (g DW d^{-1} $shoot^{-1}$)	Leaf area index
**Nov**	ZM1	12.71 ± 2.68	0.0015 ± 0.00014	10168.00 ± 1317.92
	ZM2	2.07 ± 0.9	0.00075 ± 0.00016	4104.98 ± 477.45
**Dec**	ZM1	7.55 ± 1.22	-	5277.26 ± 1663.90
	ZM2	-	-	-
**Jan**	ZM1	3.33 ± 1.76	0.00057 ± 0.00022	1099.27 ± 425.09
	ZM2	0.39 ± 0.06	0.00044 ± 0.00003	392.52 ± 97.87
**Feb**	ZM1	1.66 ± 0.97	0.00054 ± 0.00014	655.30 ± 176.67
	ZM2	0.25 ± 0.10	0.00046 ± 0.00002	267.70 ± 23.88
**Mar**	ZM1	2.73 ± 1.61	0.00070 ± 0.00023	1250.73 ± 182.81
	ZM2	0.37 ± 0.07	0.00050 ± 0.00004	364.57 ± 74.83
**Apr**	ZM1	3.68 ± 1.29	0.00076 ± 0.00023	1511.71 ± 309.17
	ZM2	0.48 ± 0.13	0.00051 ± 0.00007	394.32 ± 96.61
**May**	ZM1	5.90 ± 0.88	0.00090 ± 0.00016	2330.40 ± 235.66
	ZM2	0.85 ± 0.38	0.00060 ± 0.00011	1065.67 ± 94.07
**Jun**	ZM1	8.23 ± 3.91	0.0012 ± 0.00044	3952.03 ± 537.80
	ZM2	2.06 ± 0.65	0.00080 ± 0.0002	2614.76 ± 661.56
**Jul**	ZM1	11.93 ± 1.28	0.0013 ± 0.00077	12534.80 ± 1180.13
	ZM2	3.15 ± 1.24	0.00085 ± 0.00008	6893.47 ± 382.66
**Aug**	ZM1	11.60 ±3.06	0.0019 ± 0.00039	12808.25 ± 1212.51
	ZM2	3.31 ± 0.59	0.0011 ± 0.00014	7352.11 ± 2525.86
**Sept**	ZM1	15.46 ± 7.23	0.0021 ± 0.00047	13194.73 ± 3284.22
	ZM2	5.42 ± 2.28	0.0011 ± 0.0003	12259.82 ± 3040.03
**Oct**	ZM1	14.78 ± 1.91	0.0014 ± 0.00054	6306.09 ± 1934.05
	ZM2	2.87 ± 1.40	0.00060 ± 0.0001	3831.73 ± 1341.26

Table S3. Monthly results of carbohydrates (sucrose and starch) contents in leaves of two Irish ecotypes of *Zostera marina* (ZM1 and ZM2). Data represented as mean ± SD (n=3).

Month	Ecotype	Sucrose (mg g^{-1} DW)	Starch (mg g^{-1} DW)
**Nov**	ZM1	60.79 ± 24.67	33.36 ± 12.10
	ZM2	69.71 ± 21.59	18.10 ± 4.26
**Dec**	ZM1	37.88 ± 3.78	40.12 ± 6.40
	ZM2	6.94 ± 2.88	7.92 ± 7.2
**Jan**	ZM1	49.60 ± 2.11	55.64 ± 17.57
	ZM2	85.61 ± 8.90	28.99 ± 3.36
**Feb**	ZM1	23.31 ± 10.66	39.52 ± 6.35
	ZM2	62.29 ± 7.16	26.15 ± 0.59
**Mar**	ZM1	52.56 ± 8.19	54.93 ± 5.92
	ZM2	54.18 ± 7.69	42.01 ± 5.48
**Apr**	ZM1	51.81 ± 3.25	57.23 ± 3.28
	ZM2	80.33 ± 6.27	43.43 ± 5.77
**May**	ZM1	122.16 ± 5.80	59.13 ± 1.20
	ZM2	150.78 ± 7.35	31.74 ± 12.56
**Jun**	ZM1	69.72 ± 12.11	67.50 ± 5.90
	ZM2	100.53 ± 6.45	25.41 ± 4.30
**Jul**	ZM1	115.48 ± 3.16	66.82 ± 2.43
	ZM2	140.63 ± 6.48	26.80 ± 9.12
**Aug**	ZM1	135.78 ± 20.10	70.60 ± 3.75
	ZM2	173.29 ± 4.12	39.78 ± 14.97
**Sept**	ZM1	118.01 ± 7.10	72.10 ± 3.50
	ZM2	123.01 ± 15.23	31.05 ± 6.82
**Oct**	ZM1	123.24 ± 6.41	53.51 ± 11.79
	ZM2	106.24 ± 2.13	26.70 ± 3.26

Table S4. Monthly results of total fatty acids (TFA), monounsaturated fatty acids (MUFA), polyunsaturated fatty acids (PUFA), saturated fatty acids (SFA) contents in leaves of two Irish ecotypes of *Zostera marina* (ZM1 and ZM2). Data represented as mean ± SD (n=3-4).

%FA	Ecotype	Nov	Dec	Jan	Feb	Mar	Apr	May	Jun	Jul	Aug	Sep	Oct
**SFA**	**ZM1**	19.2 ± 0.5	19.3 ± 1.4	14.5 ± 0.4	16.8 ± 0.5	15.7 ± 0.7	16.2 ± 0.4	20.6 ± 1.0	19.7 ± 0.8	24.3 ± 1.3	21.6 ± 1.5	25.8 ± 2.1	18.8 ± 2.1
**16:0**	**ZM1**	18.3 ± 0.5	18.4 ± 1.3	13.9 ± 0.4	16.0 ± 0.5	14.9 ± 0.6	15.5 ± 0.4	19.5 ± 0.9	18.6 ± 0.8	22.9 ± 1.2	20.3 ± 1.4	24.5 ± 2.0	17.8 ± 1.3
**18:0**	**ZM1**	0.9 ± 0.0	0.8 ± 0.1	0.5 ± 0.0	0.8 ± 0.0	0.8 ± 0.1	0.7 ± 0.0	1.1 ± 0.1	1.1 ± 0.1	1.4 ± 0.1	1.3 ± 0.1	1.3 ± 0.1	1.0 ± 0.1
**MUFA**	**ZM1**	3.2 ± 0.1	3.9 ± 0.6	2.7 ± 0.1	3.3 ± 0.1	4.1 ± 0.1	4.4 ± 0.1	5.3 ± 0.2	4.1 ± 0.5	3.7 ± 0.6	4.4 ± 0.3	4.1 ± 0.6	3.8 ± 0.6
**14:1**	**ZM1**	0.7 ± 0.1	0.7 ± 0.1	0.7 ± 0.1	0.7 ± 0.1	0.6 ± 0.0	0.7 ± 0.1	0.7 ± 0.0	0.7 ± 0.0	0.8 ± 0.1	0.7 ± 0.1	0.9 ± 0.1	0.9 ± 0.1
**16:1 n-7**	**ZM1**	1.1 ± 0.1	2.1 ± 0.4	1.3 ± 0.1	1.7 ± 0.1	2.6 ± 0.1	2.6 ± 0.1	3.2 ± 0.2	2.0 ± 0.6	1.3 ± 0.4	1.8 ± 0.2	1.4 ± 0.4	1.6 ± 0.2
**18:1 n-7**	**ZM1**	0.4 ± 0.0	0.5 ± 0.1	0.3 ± 0.0	0.3 ± 0.0	0.4 ± 0.0	0.5 ± 0.0	0.6 ± 0.0	0.6 ± 0.0	0.5 ± 0.1	0.6 ± 0.1	0.6 ± 0.1	0.5 ± 0.0
**18:1 n-9**	**ZM1**	0.9 ± 0.0	0.6 ± 0.1	0.5 ± 0.0	0.5 ± 0.0	0.5 ± 0.1	0.6 ± 0.0	0.8 ± 0.1	0.8 ± 0.1	1.1 ± 0.1	1.2 ± 0.2	1.2 ± 0.1	0.8 ± 0.1
**PUFA**	**ZM1**	72.3 ± 0.6	71.5 ± 2.5	79.4 ± 0.7	75.9 ± 0.7	76.4 ± 0.6	74.6 ± 0.6	70.2 ± 1.4	72.1 ± 0.3	68.8 ± 2.2	68.5 ± 1.9	64.6 ± 3.4	72.6 ± 3.4
**16:3 n-3**	**ZM1**	9.0 ± 0.3	8.5 ± 0.9	11.3 ± 0.3	10.0 ± 0.4	10.0 ± 0.7	9.8 ± 0.5	7.1 ± 0.5	7.4 ± 0.4	5.2 ± 0.6	6.5 ± 0.7	5.1 ± 1.1	8.4 ± 0.6
**16:3 n-4**	**ZM1**	0.3 ± 0.0	0.2 ± 0.0	0.2 ± 0.0	0.2 ± 0.0	0.2 ± 0.0	0.3 ± 0.0	0.3 ± 0.0	0.6 ± 0.0	0.6 ± 0.0	0.4 ± 0.1	0.4 ± 0.0	0.3 ± 0.0
**18:2 n-6**	**ZM1**	15.0 ± 0.3	13.8 ± 0.6	11.4 ± 0.3	10.8 ± 0.1	11.0 ± 0.6	10.0 ± 0.5	13.7 ± 0.2	14.9 ± 1.7	20.0 ± 0.7	15.3 ± 0.9	16.8 ± 0.5	14.6 ± 0.6
**18:3 n-3**	**ZM1**	48.1 ± 0.3	49.0 ± 2.2	56.6 ± 0.4	55.0 ± 0.6	55.3 ± 0.7	54.5 ± 0.4	49.1 ± 0.9	49.1 ± 1.2	42.9 ± 1.1	46.3 ± 2.0	42.3 ± 2.3	49.3 ± 1.9
**Others**	**ZM1**	5.3 ± 0.3	5.4 ± 0.7	3.4 ± 0.3	4.0 ± 0.2	3.7 ± 0.1	4.8 ± 0.5	3.9 ± 0.3	4.1 ± 0.3	3.2 ± 0.4	5.5 ± 0.7	5.5 ± 1.0	4.8 ± 0.5
**TFA**	**ZM1**	2.6 ± 0.1	2.7 ± 0.2	2.9 ± 0.0	2.7 ± 0.1	2.8 ± 0.2	2.8 ± 0.2	1.8 ± 0.2	2.4 ± 0.1	1.5 ± 0.1	1.8 ± 0.1	1.5 ± 0.2	2.3 ± 0.1
**PUFA/SFA**	**ZM1**	3.8 ± 0.1	3.7 ± 0.4	5.5 ± 0.2	4.5 ± 0.2	4.9 ± 0.3	4.6 ± 0.1	3.4 ± 0.2	3.7 ± 0.2	2.8 ± 0.2	3.2 ± 0.3	2.5 ± 0.4	3.9 ± 0.4
**Omega 3/6**	**ZM1**	3.8 ± 0.1	4.2 ± 0.4	6.0 ± 0.2	6.0 ± 0.1	5.9 ± 0.5	6.4 ± 0.3	4.1 ± 0.1	3.9 ± 0.6	2.4 ± 0.1	3.5 ± 0.4	2.8 ± 0.2	4.0 ± 0.3
**18:3 n-3/16:0**	**ZM1**	2.6 ± 0.1	2.7 ± 0.3	4.1 ± 0.1	3.4 ± 0.1	3.7 ± 0.2	3.5 ± 0.1	2.5 ± 0.2	2.6 ± 0.2	1.9 ± 0.1	2.3 ± 0.3	1.7 ± 0.3	2.8 ± 0.3
**SFA**	**ZM2**	20.0 ± 0.5	19.5 ± 0.6	15.0 ± 0.3	17.8 ± 0.8	16.3 ± 0.2	16.5 ± 0.5	16.4 ± 0.2	19.8 ± 0.9	21.4 ± 0.4	24.9 ± 1.1	21.6 ± 1.7	17.0 ± 0.0
**16:0**	**ZM2**	19.0 ± 0.5	18.7 ± 0.6	14.4 ± 0.2	17.0 ± 0.8	15.5 ± 0.2	15.7 ± 0.5	15.6 ± 0.2	18.6 ± 0.9	20.1 ± 0.4	23.4 ± 1.1	20.5 ± 1.6	16.2 ± 0.1
**18:0**	**ZM2**	1.0 ± 0.0	0.9 ± 0.0	0.6 ± 0.0	0.8 ± 0.0	0.7 ± 0.0	0.8 ± 0.0	0.9 ± 0.0	1.3 ± 0.1	1.3 ± 0.0	1.5 ± 0.0	1.1 ± 0.1	0.8 ± 0.1
**MUFA**	**ZM2**	4.1 ± 0.2	4.3 ± 0.3	2.6 ± 0.1	2.5 ± 0.4	2.7 ± 0.2	2.8 ± 0.2	2.4 ± 0.2	2.3 ± 0.2	2.5 ± 0.2	3.9 ± 0.2	3.8 ± 0.3	1.9 ± 0.2
**14:1**	**ZM2**	1.1 ± 0.0	0.8 ± 0.1	0.8 ± 0.1	0.8 ± 0.0	0.7 ± 0.0	0.6 ± 0.2	0.4 ± 0.0	0.5 ± 0.0	0.4 ± 0.1	0.5 ± 0.1	0.5 ± 0.1	0.4 ± 0.1
**16:1 n-7**	**ZM2**	1.7 ± 0.3	2.2 ± 0.2	0.8 ± 0.1	0.7 ± 0.4	0.9 ± 0.1	1.1 ± 0.2	0.8 ± 0.2	0.4 ± 0.1	0.7 ± 0.2	1.4 ± 0.1	1.7 ± 0.2	0.6 ± 0.1
**18:1 n-7**	**ZM2**	0.5 ± 0.0	0.5 ± 0.0	0.3 ± 0.0	0.4 ± 0.0	0.4 ± 0.0	0.4 ± 0.0	0.5 ± 0.0	0.5 ± 0.0	0.5 ± 0.1	0.7 ± 0.0	0.6 ± 0.0	0.4 ± 0.0
**18:1 n-9**	**ZM2**	1.0 ± 0.1	0.8 ± 0.0	0.6 ± 0.0	0.8 ± 0.2	0.6 ± 0.0	0.8 ± 0.1	0.6 ± 0.1	0.9 ± 0.1	0.8 ± 0.1	1.3 ± 0.1	0.9 ± 0.1	0.5 ± 0.0
**PUFA**	**ZM2**	72.9 ± 1.0	72.7 ± 0.9	80.0 ± 0.5	76.6 ± 1.1	78.4 ± 0.4	78.4 ± 1.0	79.2 ± 0.1	75.5 ± 1.0	73.8 ± 0.5	68.2 ± 1.4	71.4 ± 2.2	78.5 ± 0.3
**16:3 n-3**	**ZM2**	8.5 ± 0.5	8.1 ± 0.2	10.5 ± 0.2	9.7 ± 0.6	10.0 ± 0.1	9.9 ± 0.2	9.0 ± 0.2	8.0 ± 0.4	6.9 ± 0.2	5.4 ± 0.4	7.2 ± 0.6	9.9 ± 0.1
**16:3 n-4**	**ZM2**	1.6 ± 0.2	2.1 ± 0.0	1.6 ± 0.1	2.1 ± 0.2	2.9 ± 0.1	2.6 ± 0.2	1.9 ± 0.1	1.7 ± 0.4	1.5 ± 0.1	2.2 ± 0.2	1.5 ± 0.1	1.3 ± 0.1
**18:2 n-6**	**ZM2**	14.9 ± 0.3	14.2 ± 0.3	12.1 ± 0.1	11.7 ± 0.5	10.7 ± 0.2	11.1 ± 0.4	12.2 ± 0.3	15.2 ± 0.9	16.9 ± 0.4	15.9 ± 0.5	14.6 ± 0.4	14.4 ± 0.2
**18:3 n-3**	**ZM2**	47.9 ± 0.6	48.3 ± 0.7	55.8 ± 0.5	53.2 ± 1.2	54.8 ± 0.3	54.8 ± 1.2	56.2 ± 0.1	50.6 ± 1.1	48.5 ± 0.7	44.7 ± 1.6	48.0 ± 1.9	52.8 ± 0.4
**Others**	**ZM2**	3.0 ± 0.3	3.5 ± 0.1	2.4 ± 0.2	3.0 ± 0.2	2.7 ± 0.1	2.3 ± 0.4	2.0 ± 0.1	2.3 ± 0.1	2.3 ± 0.2	3.0 ± 0.1	3.2 ± 0.2	2.6 ± 0.1
**TFA**	**ZM2**	2.6 ± 0.1	2.5 ±0.1	2.7 ± 0.1	2.4 ± 0.5	2.7 ± 0.1	2.7 ± 0.1	2.1 ± 0.1	2.4 ± 0.2	1.8 ± 0.1	1.6 ± 0.1	2.1 ± 0.2	2.8 ± 0.2
**PUFA/SFA**	**ZM2**	3.7 ± 0.1	3.7 ± 0.2	5.3 ± 0.1	4.3 ± 0.2	4.8 ± 0.1	4.8 ± 0.2	4.8 ± 0.1	3.8 ± 0.2	3.5 ± 0.1	2.8 ± 0.2	3.3 ± 0.4	4.6 ± 0.0
**Omega 3/6**	**ZM2**	3.8 ± 0.0	4.0 ± 0.1	5.5 ± 0.1	5.4 ± 0.4	6.1 ± 0.1	5.8 ± 0.3	5.3 ± 0.1	3.9 ± 0.3	3.3 ± 0.1	3.2 ± 0.2	3.8 ± 0.2	4.3 ± 0.1
**18:3 n-3/16:0**	**ZM2**	2.5 ± 0.1	2.6 ± 0.1	3.9 ± 0.1	3.1 ± 0.2	3.5 ± 0.1	3.5 ± 0.2	3.6 ± 0.0	2.7 ± 0.2	2.4 ± 0.1	1.9 ± 0.2	2.4 ± 0.2	3.3 ± 0.0

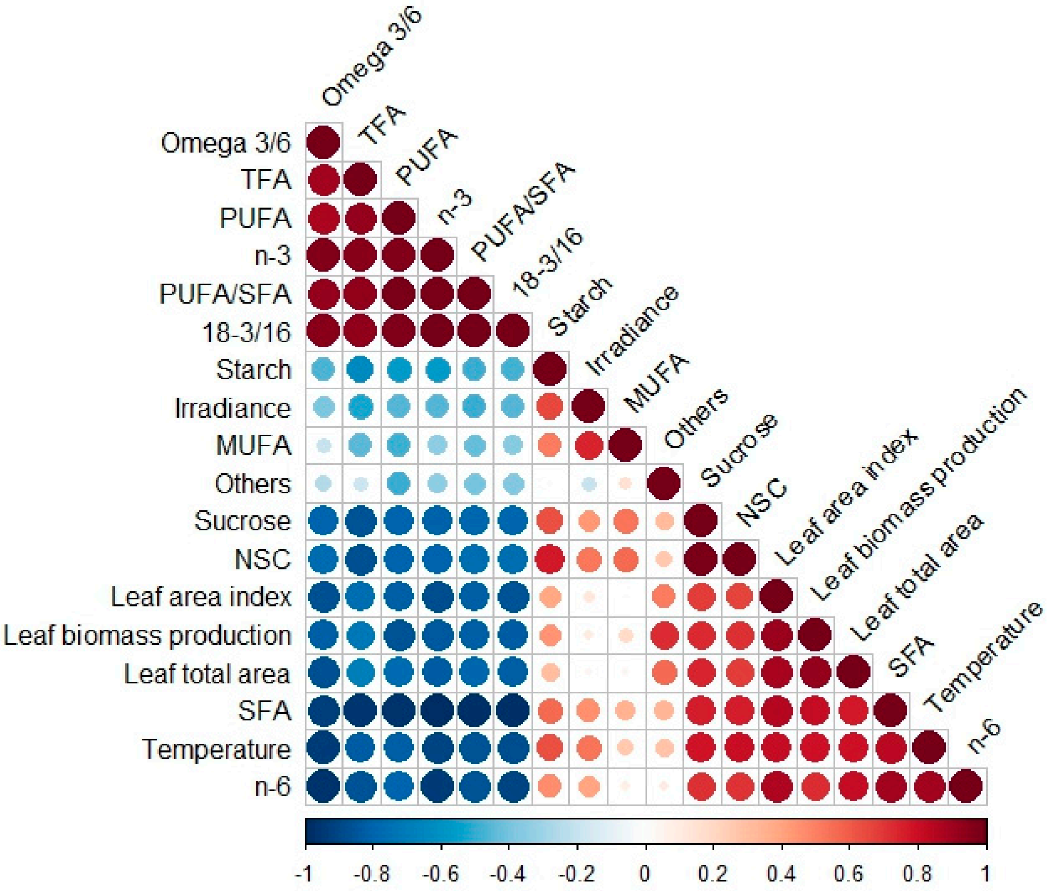

Figure S2. *Z. marina* ecotype (ZM1) in Galway Bay. Correlation matrix based on person Pearson's coefficient correlating seagrass descriptors, irradiance and SST. Positive correlations are represented in red and negative in blue. The higher size of the circles indicates a higher correlation between the compared variables (n=12).

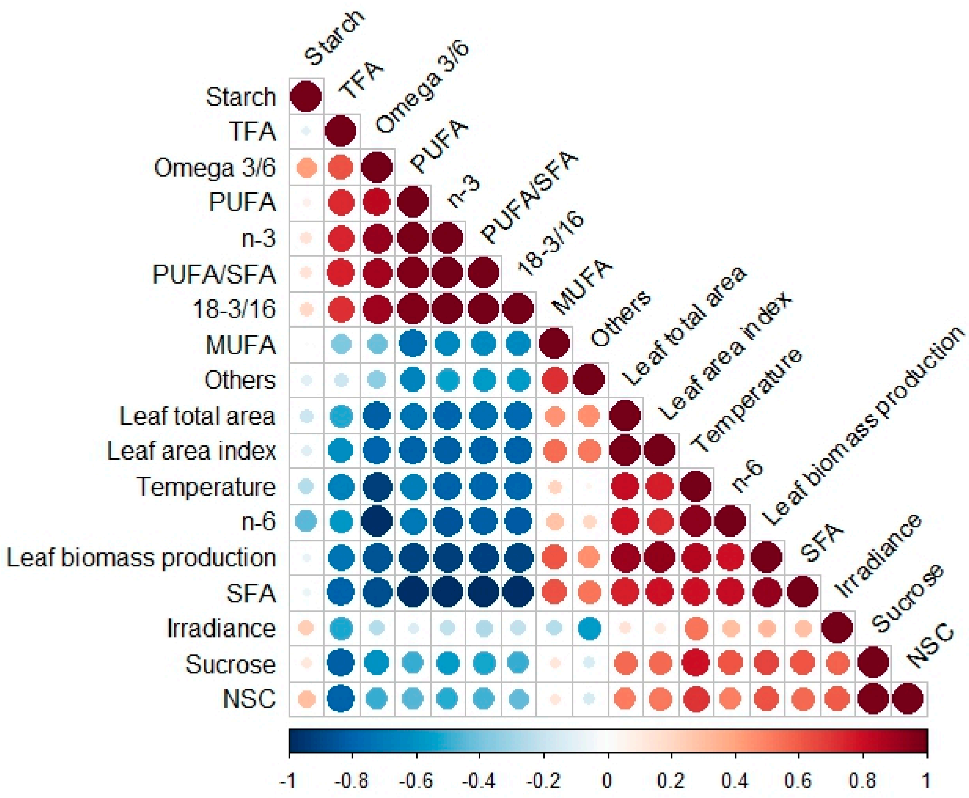

Figure S3. *Z. marina* ecotype (ZM2) in Galway Bay. Correlation matrix based on person Pearson's coefficient correlating seagrass descriptors, irradiance and SST. Positive correlations are represented in red and negative in blue. The higher size of the circles indicates a higher correlation between the compared variables (n=12).

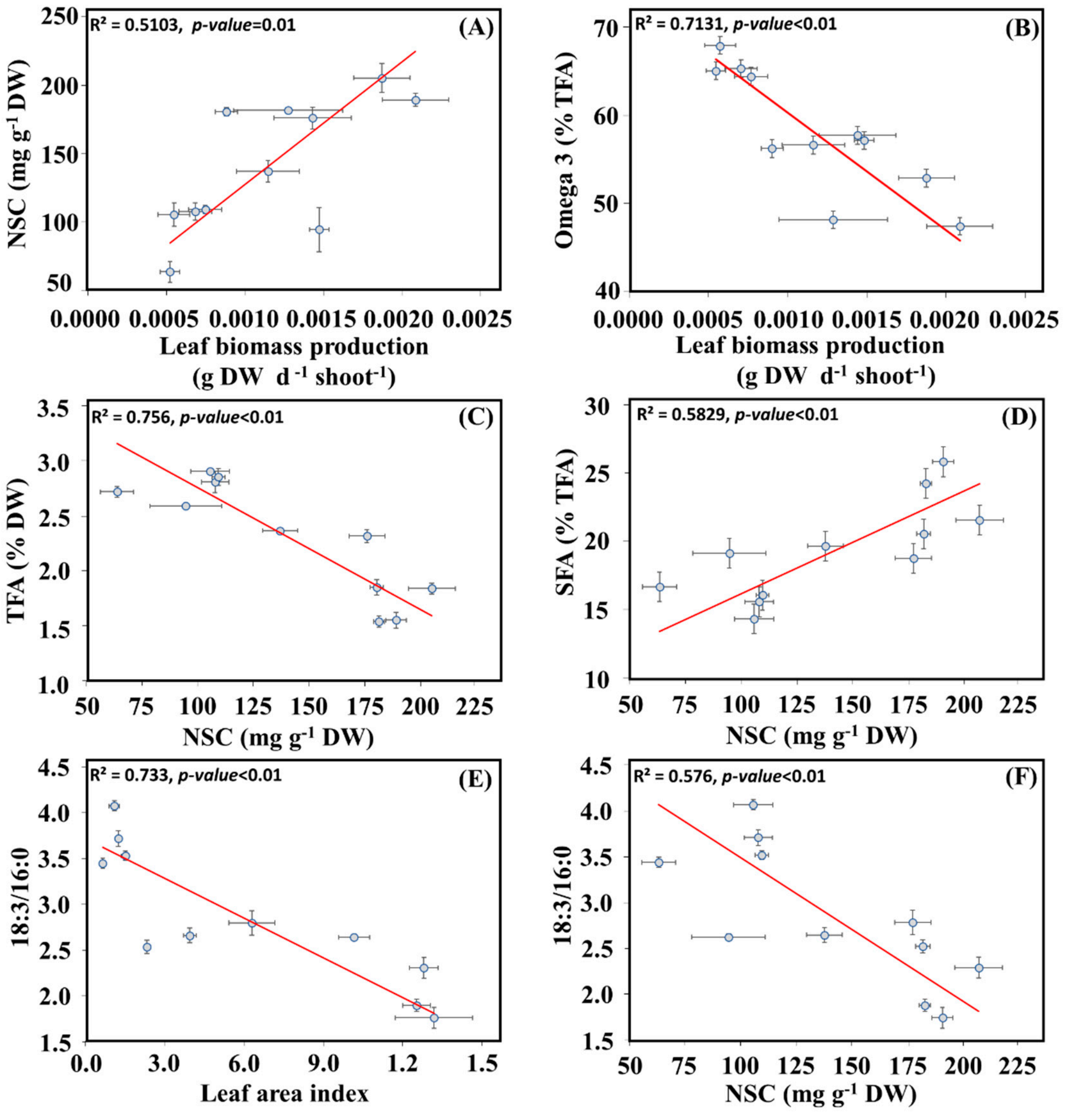

Figure S4. *Z. marina* ecotype (ZM1) in Galway Bay. Deming correlation of non-structural carbohydrates (NSC) *versus* leaf biomass production (Panel A); omega 3 (n-3 PUFA) *versus* leaf biomass production (Panel B); total fatty acids (TFA) *versus* non-structural carbohydrates (NSC) (Panel C); saturated fatty acids (SFAs) *versus* non-structural carbohydrates (NSC) (Panel D); 18:3/16:0 ratio *versus* leaf area index (Panel E); 18:3/16:0 ratio *versus* non-structural carbohydrates (NSC) (Panel F). Red lines represent the Deming regression line (n=12).

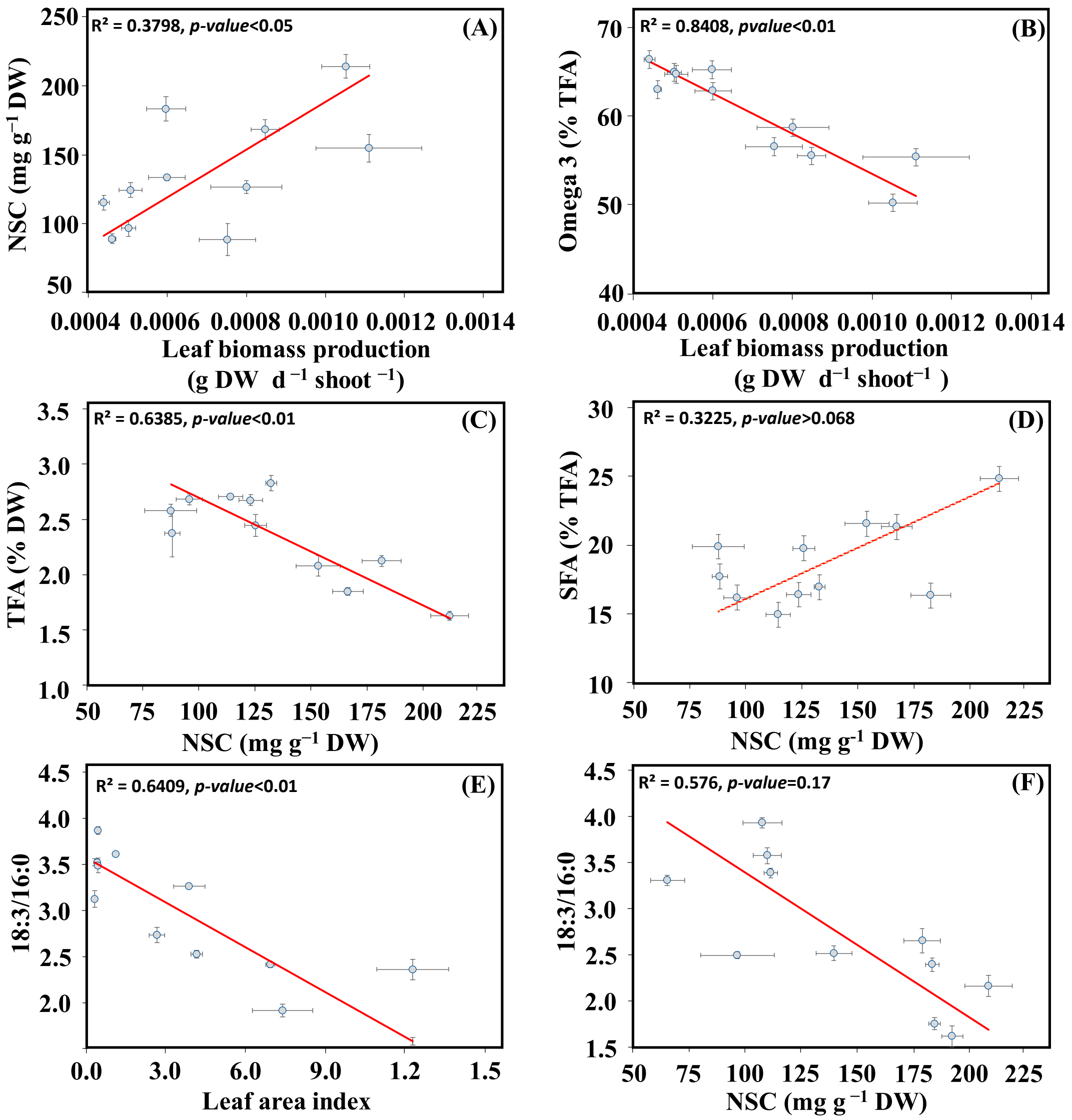

Figure S5. *Z. marina* ecotype (ZM2) in Galway Bay. Deming correlation of non-structural carbohydrates (NSC) *versus* leaf biomass production (Panel A); omega 3 (n-3 PUFA) *versus* leaf biomass production (Panel B); total fatty acids (TFA) *versus* non-structural carbohydrates (NSC) (Panel C); saturated fatty acids (SFAs) *versus* non-structural carbohydrates (NSC) (Panel D); 18:3/16:0 ratio *versus* leaf area index (Panel E); 18:3/16:0 ratio *versus* non-structural carbohydrates (NSC) (Panel F). Red lines represent the Deming regression line (n=12).
